# Comprehensive Analysis of Characteristics of Cuproptosis-Related LncRNAs Associated with Prognosis of Lung Adenocarcinoma and Tumor Immune Microenvironment

**DOI:** 10.3390/ph17091244

**Published:** 2024-09-21

**Authors:** Feihong Chen, Xin Wen, Jiani Wu, Min Feng, Shicheng Feng

**Affiliations:** 1 Pharmaceutical Research Center, School of Chemistry and Chemical Engineering, Southeast University, Nanjing 211189, China; chenfeihong@seu.edu.cn (F.C.); wenx9911@163.com (X.W.); 220233122@seu.edu.cn (J.W.); 2 Department of Radiotherapy, Zhongda Hospital, Southeast University, Nanjing 210009, China; 18012965164@163.com; 3 School of Medicine, Southeast University, Nanjing 210009, China

**Keywords:** lung adenocarcinoma (LUAD), immune microenvironment, cuproptosis, lncRNA, prognostic markers, drug sensitivity

## Abstract

As a novel discovered mechanism of cell death, cuproptosis is copper-dependent and induces protein toxicity related to advanced tumors, disease prognosis, and human innate and adaptive immune response. However, it has not yet been fully established how the prognosis of lung adenocarcinoma (LUAD) is related to the immune microenvironment of cuproptosis-related lncRNAs using several bioinformatic techniques. In the study, 19 genes related to cuproptosis were collected. Subsequently, 783 lncRNAs related to the co-expression of cuproptosis were obtained. Moreover, the Cox model revealed and constructed four lncRNA (*AC012020.1*, *AC114763.1*, *AL161431.1*, *AC010260.1*) prognostic markers related to cuproptosis. Based on the median risk score (RS) values, patients were categorized into two groups: high risk and low risk. The Kaplan–Meier (KM) survival curve depicted a statistically significant overall survival (OS) rate among two groups. Principal component analysis (PCA) and receiver operator characteristic curve (ROC) proved that the model had promising ability in prognosis. The analysis of univariate and multivariate Cox regression revealed that RS served as an independent prognostic factor. Moreover, multivariate Cox regression was employed for the establishment of a nomogram of prognostic indicators. The tumor mutational burden (TMB) depicted a considerable difference between the two risk groups. The immunotherapy response of LUAD patients with high risk was improved compared to low risk patients. The study also revealed that drug sensitivity associated with LUAD was significantly linked to RS. The findings could be helpful to establish a good diagnosis, prognosis, and management regime for patients with LUAD.

## 1. Introduction

Lung adenocarcinoma (LUAD) is a malignant tumor jeopardizes human health to a great extent. Though some treatment strategies are available, such as surgery, chemotherapy, radiotherapy, and immunotherapy, the risk of recurrence and metastasis is still high. New diagnosis and treatment methods are urgently needed to meet the challenge of radical cancer treatment to improve the five-year survival rate of LUAD patients. Considering dual functions, copper is an essential enzyme cofactor that can result in cell death. It is reported that copper participates in the novel cell death process of cuproptosis. Cuproptosis is a variant of common cell death mechanisms including apoptosis, ferroptosis, and autophagy. It can cause cell death using copper ions when these other mechanisms do not work [1]. Therefore, copper is being studied as a new target for treatments to explore its potential in anti-tumor therapy. Based on this cell death method, novel therapeutic strategies are being developed for LUAD patients. Therefore, determining the key regulatory factors responsible for cuproptosis is essential to further understand how the process works.

Long non-coding RNA (LncRNA) is considered as an mRNA transcript that is more than 200 nucleotides long. These nucleotides are extracted from non-protein-encoding corresponding genes [2]. Using epigenetic and post-translational mechanisms, modification, transcriptional regulation, and RNA protein stability regulation, lncRNAs can be considered as vital regulators of biological processes, unlike mRNA, which only functions in protein translation [3]. Increasing evidence shows that lncRNA is a key regulator of the pathogenesis, progress, and metastasis of a wide range of cancers [4,5]. Many lncRNAs are promising biomarkers for cancer diagnosis and prognosis, including endometrial, gastric, and lung cancers [6,7,8]. The role of lncRNA in regulating cuproptosis has been determined by a limited number of studies, which is why it is necessary to further explore its role and potential in the prognosis prediction of LUAD patients [9,10]. In the study, data downloaded from TCGA was obtained, and two groups for training and validation were developed by randomly allocating patients. The prognostic markers of lncRNAs related to cuproptosis were determined. A predictive model of lncRNA characteristics was developed to determine promising treatment targets and lay the basis for effective clinical utility and prognostic determination of LUAD patients. Finally, the model’s prognostic efficiency was verified to establish effective therapeutic strategies and reveal its predictive ability regarding immunotherapy and drug sensitivity as associated with LUAD. The study was conducted to explore the prognosis-related lncRNA markers of cuproptosis to improve the current diagnosis, treatment, and prevention strategies of LUAD.

## 2. Results

### 2.1. Data Processing

**Figure 1:**
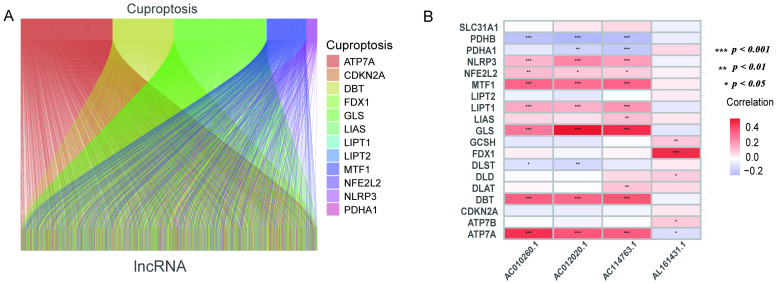
Sankey diagram and heat map. (**A**) Sankey diagram of co-expression between 19 cuproptosis-related genes and 240 cuproptosis-related lncRNAs. (**B**) Correlation of 19 cuproptosis-related genes and 4 prognostic cuproptosis-related lncRNAs. * *p* < 0.05, ** *p* < 0.01, and *** *p* < 0.001.

The gene annotation information was obtained based on the “GENCODE” database, and 13,986 lncRNAs were identified in the TCGA_LUAD data set after removing the genes with protein-coding functions. A total of 19 genes related to cuproptosis were collected. A total of 240 cuproptosis-related lncRNAs were extracted using Pearson analysis. The Sankey diagram reveals the linkages between cuproptosis-related genes and lncRNA (Figure 1A and Supplementary Materials S2). After that, univariate Cox regression analysis helped in the exploration of lncRNA associated with cuproptosis (*p* < 0.05). A total of 445 patients were categorized into a training set (*n* = 223) and a validation set (*n* = 222). Table 1 depicts the clinical data of LUAD. The findings indicated no difference between the clinical data of the two groups.

### 2.2. Construction and Validation of Prognostic Markers of Cuproptosis-Related LncRNA

**Figure 2:**
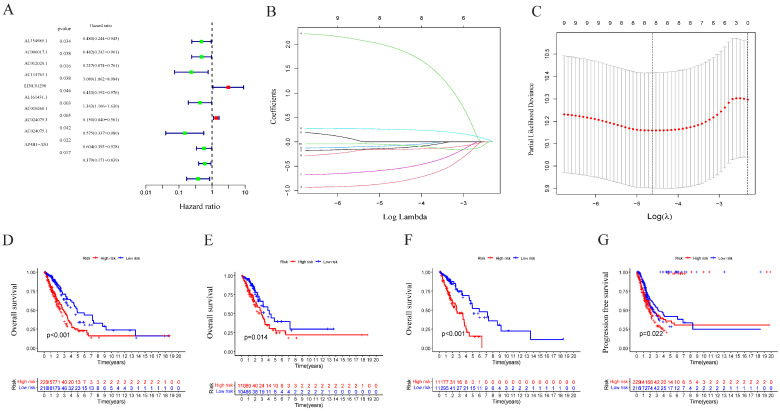
Construction of the prognostic cuproptosis-related lncRNA risk model in LUAD. (**A**) Univariate Cox regression analysis for identifying the prognostic cuproptosis-related lncRNAs. (**B,C**) Lasso–Cox regression analysis was performed to construct prognostic prediction models. (**D**) Kaplan–Meier curves for survival analysis in the high- and low-risk groups in the entire cohort. (**E**) Kaplan–Meier curves for survival analysis in the validation set. (**F**) Kaplan–Meier curves for survival analysis in the training set. (**G**) Kaplan–Meier curves of progression-free survival (PFS).

**Figure 3:**
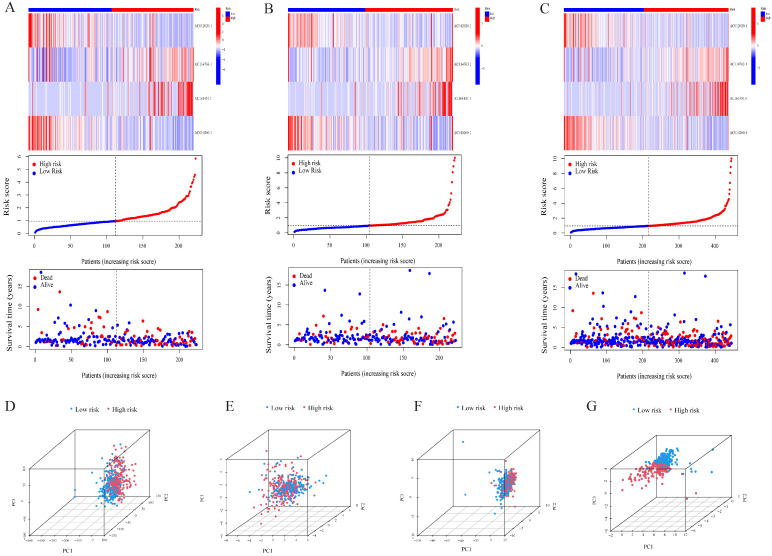
Validation of the risk model in the cohort and principal component analysis. Risk score distribution, survival status, and heatmap of the prognostic markers and overall survival in the cohort. (**A**) The training set. (**B**) The validation set. (**C**) The entire cohort. PCA between the high- and low-risk groups based on (**D**) all genes, (**E**) cuproptosis-related genes, (**F**) cuproptosis-related lncRNAs, and (**G**) cuproptosis-related lncRNA prognostic markers.

The univariate Cox analysis of 10 lncRNAs associated with cuproptosis is presented in Figure 2A. Furthermore, eight lncRNAs were chosen with the help of Lasso Cox regression. The track changes of the lncRNA regression coefficient and the results obtained after cross-validating the construction model were determined (Figure 2B,C). After that, multivariate Cox regression analysis was conducted for the identification of four prognosis-related lncRNAs and the establishment of an RS model. In conclusion, a total of four lncRNAs were generated to construct a predictive prognostic model of OS for LUAD patients. Subsequently, creating a heat map determined the relationship between cuproptosis-related genes and lncRNA (Figure 1B). RS = (−1.2474111527 × Expression AC012020.1) + (2.228145456 × Expression AC114763.1) + (0.276899619759326 × Expression AL161431.1) + (−1.001918665 × Expression AC010260.1); according to median RS, OS and PFS of patients in two groups were analyzed using the KM survival curve (Supplementary Materials S3/S4). Before the survival analysis, all patients were divided into two risk groups of high values and low values. The findings indicated a decreased OS for high risk patients and an increased OS for low risk patients (*p* < 0.05; Figure 2D). A notable difference was observed in the OS of risk groups in the validation and training sets (*p* < 0.05; Figure 2E,F). A notable difference in PFS was observed in the two risk groups (*p* < 0.05, Figure 2G). The distribution of RS and patient survival status is depicted in the data of Figure 3. The heat map shows the expression levels of four lncRNAs related to death, including both risk sets. An increase in the value of RS showed a prominent decrease in survival time, and as a result, the death count increased (Figure 3A–C). PCA exhibited a significant variation among the two risk groups. Based on cuproptosis-related lncRNA, LUAD patients were categorized into two groups (Figure 3D–G).

### 2.3. Independence of Prognostic Markers of Cuproptosis-Related LncRNAs in Predicting Overall Survival

**Figure 4:**
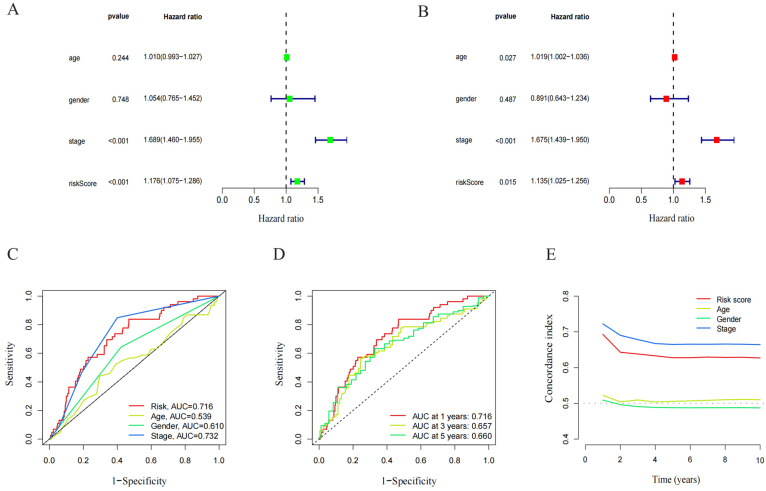
Independent prognostic analysis of LUAD overall survival (OS). (**A**) Univariate Cox analysis. Age, stage, and risk score were statistically significant. (**B**) Multivariate Cox analysis. Age, stage, and risk score were statistically significant. (**C**) ROC demonstrated the predictive accuracy of the risk model. (**D**) Time ROC curve predicted 1, 3, and 5 years of OS for LUAD patients. (**E**) C-index showed the predictive accuracy of the risk model and stage was superior to other clinical parameters.

**Figure 5:**
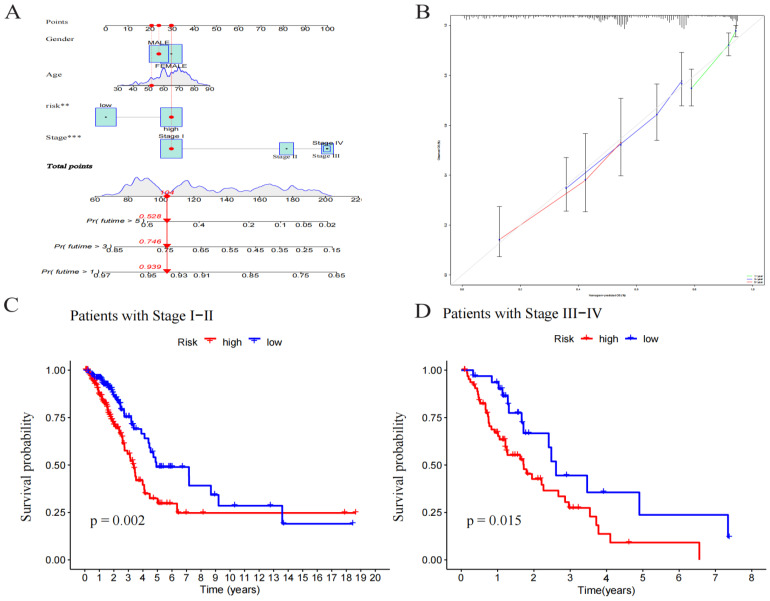
Construction and evaluation of a nomogram based on lncRNAs. (**A**) A nomogram used to predict prognosis was constructed based on lncRNAs. ** *p* < 0.01, and *** *p* < 0.001. (**B**) Calibration curves are used to predict 1-, 3-, and 5-year overall survival. (**C**) Kaplan–Meier curves of patients with stage I–II. (**D**) Kaplan–Meier curves of patients with stage III–IV.

The prognostic model’s predictive value was assessed using univariate and multivariate Cox regression analysis. In the univariate Cox analysis, there was a statistical difference between stage and RS (Figure 4A). In the multivariate Cox regression analysis, there was still an OS predictive value (Figure 4B), while in univariate and multivariate analysis, age and gender were unrelated to prognosis. The ROC depicted the similarity of the prediction accuracy of the prognosis model with the clinical stage and its superiority over other clinical indicators such as age and gender (Figure 4C). It also proved the precision and diagnostic efficiency of cuproptosis-related lncRNA for OS. The value of AUC was 0.716, 0.657, and 0.660 for one, three, and five years, respectively (Figure 4D). The C index is indicative of the better prognostic accuracy of the model as compared to that of age and sex (Figure 4E). A prognostic nomogram was constructed depending upon RS and other clinical features (Figure 5A). The calibration chart shows improved consistency regarding the nomogram predictions (Figure 5B). Furthermore, to study the clinical efficacy of cuproptosis-related lncRNA, the relationship between cuproptosis-related lncRNA and clinical features was explored. The findings depicted a notable variation in the distribution of both RS and clinical stages. Specifically, the I–II and III–IV stages were statistically significant (Figure 5C,D).

### 2.4. Pathway and Gene Enrichment Analyses

**Figure 6:**
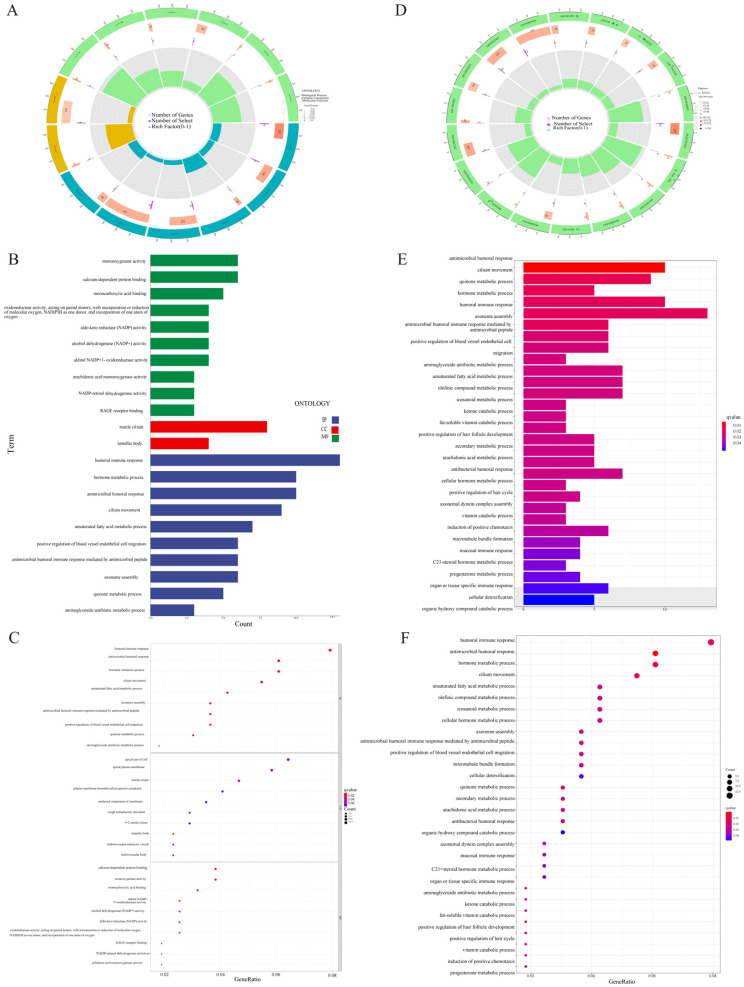
Gene Ontology (GO) and Kyoto Encyclopedia of Genes and Genomes (KEGG) pathway enrichment analysis. (**A**) Circle diagram of GO enrichment analysis. (**B**) Bar plot of the top 10 GO enrichment terms. (**C**) Bubble chart of the top 10 GO enrichment terms. (**D**) Circle diagram of KEGG enrichment analysis. (**E**) Bar plot of the top 30 KEGG enrichment terms. (**F**) Bubble chart of the top 30 KEGG enrichment terms.

**Figure 7:**
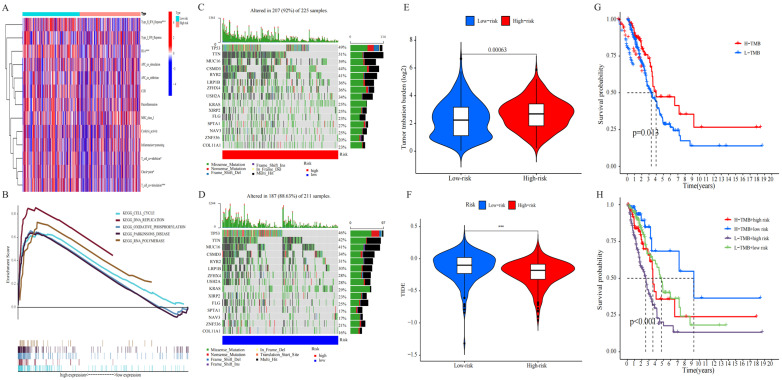
Immunological landscape in LUAD patients and relationship between tumor mutation burden (TMB) and risk score. (**A**) Heatmap of the tumor-infiltrating lymphocytes based on single-sample gene set enrichment analysis algorithms among the high- and low-risk groups in LUAD. (**B**) Multi-GSEA showed the enrichment pathway in high-risk groups. (**C**) Waterfall plot of top 15 mutant genes in the high-risk group in LUAD. (**D**) Waterfall plot of top 15 mutant genes in the low-risk group in LUAD. (**E**) Analysis of TMB differences between the high- and low-risk groups in LUAD. (**F**) Comparison of TIDE prediction score between the high- and low-risk groups. (**G**) Survival analysis curves of the high- and low-TMB groups. (**H**) TMB risk combined with survival curve in LUAD.

GO and KEGG enrichment analyses were further used for evaluation of the DEGs of both risk groups. A total of 254 DEGs were selected. Genes mainly focus on the biological processes of humoral immune response, hormone metabolism, and antibacterial humoral response. In terms of cellular components, they are mainly enriched in active cilia and layered bodies. In the molecular functions category, they regulate monooxygenase activity, calcium-dependent protein binding, and monocarboxylic acid binding (Figure 6A–C). KEGG analysis depicted the enrichment of the gene dominantly in antibacterial humoral reaction, ciliary movement, and quinone metabolism (Figure 6D,E). Multi-GSEA showed that the enrichment pathway related to high risk groups was mainly involved in cell cycles, DNA repair, redox, Parkinson’s disease, and RNA methylation (Figure 7B). No prominent variations were observed in the signaling pathways of the low risk group.

### 2.5. Estimation of Immune Cell Infiltration and Immunotherapy in Tumors

The heat map of the immune reactivity was obtained using the ssGSEA algorithm as a basis. The correlation analysis between immune cell population and related functions revealed that Type_II_IFN_Reponse, HLA/T_cell_co-inhibition, and Check-point/T_cell_co-stimulation had a prominent variation between the two risk groups (*p* < 0.05; Figure 7A). The findings indicated the relation of cuproptosis-related lncRNA with immune cell infiltration in LUAD.

### 2.6. Analysis of TMB

The somatic mutation data of LUAD were collected, and the respective TMB score was determined to study the apparent tumor mutation and its correlation with the LUAD survival rate. The findings indicated that high-risk groups are more likely to own gene mutations. TP53, TTN, MUC16, CSMD3, and RYR2 were the five genes that showed the most mutation in both risk groups as presented in Figure 7C,D, and mutation rate was even higher in the high-risk group (Figure 7E). Simultaneously, the relationship between TMB and RS was discussed. The survival analysis was conducted following the allocation of patients into two TMB groups of high and low values, depending on the median cutoff point. As shown in Figure 7G, high-risk patients had a higher TMB with an improved OS (*p* = 0.013), and low-risk patients had a lower TMB in LUAD. It should be noted that patients with low TMB and high RS had a poorer prognosis than patients with high TMB or low-risk scores, RS *(p* < 0.001) (Figure 7H). The results were indicative of the significant correlation of this risk model with the TMB of LUAD, and the combination of RS and TMB helped predict the LUAD prognosis. The immunotherapeutic effect on patients was checked using the TIDE algorithm. Figure 7F shows the significant difference in the TIDE scores among both groups, the high-risk groups have lower TIDE scores, indicating that high-risk patients are less likely to escape from the immune system and might achieve better effects with immunotherapy (Supplementary Materials S5).

### 2.7. Drug Sensitivity Analysis

**Figure 8:**
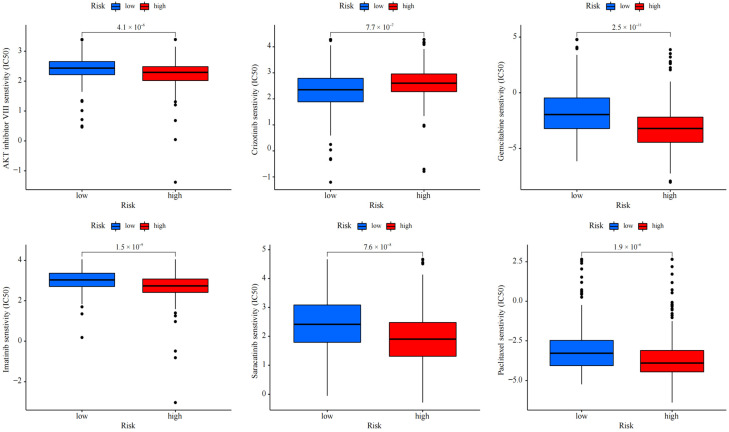
Drug effectiveness of different risk groups.

Depending on the above findings, for the purpose of determining the potential of cuproptosis-related lncRNA in LUAD for individualized treatment, the association of drug RS with IC_50_ was explored. The sensitivity of 50 common anti-cancer drugs between both groups was compared. The findings indicated six drugs (Figure 8), and the sensitivity of most anti-cancer drugs among both groups was notably variant (*p* < 0.05). The IC_50_ of five drugs in the high-risk group was lower, proving that high-risk patients were more likely to show drug sensitivity. This determined the promising potential of these drugs in LUAD treatment. However, the IC_50_ of crizotinib was higher in the high-risk patients, indicating that the low-risk patients were more sensitive to the drug. The findings exhibited that in LUAD patients, besides crizotinib, the RS was negatively correlated with IC_50_, which means that our risk characteristics can be used as an indicator to predict chemical sensitivity.

## 3. Discussion

“Cuproptosis”, a newly identified death mode, depends on the induction and regulation of copper ions. It is different from the apoptosis pathway but similar to ferroptosis. In the tricarboxylic acid cycle, copper ions induce the aggregation of lipoacylated proteins and down-regulate the expression of iron-sulfur cluster proteins, thus inducing protein toxic stress and inducing cell death [1]. Prior research indicated the tumor’s existence, and progression might be related to the imbalance of copper homeostasis. The content or distribution of copper ions in different cancer tissues, including breast and cervical cancer, is increased or abnormal, and cancer cells with strong metabolism may have a higher demand for copper [11,12]. The discovery of cuproptosis helps us to understand further copper metabolic diseases and their potential molecular mechanisms, which can serve as a promising method of treating cancer. Simultaneously, lncRNA biologically impacts cancer growth and treatment. In recent years, many studies have emphasized establishing the characteristics of coding genes and non-coding RNAs to assess cancer prognosis. LncRNA’s role in LUAD prognosis and its potential for use as a therapeutic target has been established. However, its linkage to cuproptosis-related mechanisms remains unclear.

During this research, eight cuproptosis-related lncRNAs related to LUAD prognosis were identified, four of which were selected to construct the prognosis prediction of patients. Initially, several cuproptosis-related genes and cuproptosis-related lncRNAs were selected. Following this, Lasso and Cox regression was utilized for determining the prognosis-associated lncRNA. Moreover, the correlation between lncRNA in LUAD and the common clinical features, upstream regulation mechanism, infiltration of immune cells, immunotherapy, and sensitivity of drugs were determined. On the basis of univariate Cox, Lasso, and multivariate Cox regression analysis, four prognosis-related lncRNAs were determined. LncRNA has anti-tumor or carcinogenic functions. Lai et al. found that *AC012020.1* is also a ferroptosis-related lncRNA, a biomarker of gastric cancer prognosis. Unlike other lncRNAs, patients with gastric cancer with high expression of *AC012020.1* and *AC243829.4* have a higher survival rate [13]. It is reported that human lncRNA sequences including *AC012020.1* and SARS-CoV-2 genes have nucleotide complementarities, which may be involved in destroying the epigenetic control of target genes [10]. A few studies have been conducted on *AC114763*’s involvement in the genetic susceptibility of asthma in children, but the mechanism that lncRNA *AC114763* may participate in has not been specifically clarified [14]. LncRNA AL161431.1 has been reported to be involved in the pathogenesis of different cancers. In endometrial cancer, *AL161431.1*, as an oncogene, promotes cell proliferation and migration through the MAPK signal pathway [6]. Through constructing a ceRNA network related to survival, it was found that AL161431.1 had a significant predictive value for the OS of lung squamous cell carcinoma (LUSC) patients [15]. Shao et al. identified the upregulation of *AL161431.1* expression levels under hypoxic conditions [16]. In vitro analysis showed that *AL161431.1* might play a regulatory role by combining with miR-1252-5p, and inhibiting *AL161431.1* would restrict cell growth and the transport of A549 cells and induce apoptosis. Song found that *AL161431.1* is increasingly expressed in non-small-cell lung cancer tissue, which is unfavorable for the prognosis of patients, coinciding with the findings of this research [7]. Similarly, *AC010260.1*, as an m6A-related lncRNA, participates in the differentiation of cluster construction with survival differences, which can also help in anticipating the clinical progress and prognosis risk associated with LUAD [17]. Subsequently, the risk model’s predictive accuracy was verified. The KM method identified a high OS of low-risk patients and vice versa. The ROC curve showed that cuproptosis-related lncRNA has high accuracy in predicting one-, three-, and five-year survival rates. The AUC was greater than 0.65, and one- and three-year prediction performance was better than in previous studies [18]. Combined with cuproptosis-related lncRNA and clinical information, a new nomogram was developed for the prognosis prediction of LUAD. The model had a similar prediction ability with clinical stages, providing a basis for the prediction of patients with unclear clinical stages. The findings showed that TMB in the high-risk group was statistically higher compared to the low-risk group, proving that LUAD high-risk patients respond better to immunotherapy. Predicting cuproptosis-related features based on the tumor microenvironment status of LUAD may help clarify the TME mechanism of cuproptosis in LUAD and explore more effective immunotherapy strategies for LUAD. TP53 and TTN were the most mutated genes in LUAD patients. In addition, analyzing the drug sensitivity of CRPLM can pave the way for effective clinical treatment. Notable variations were observed in the IC_50_ of all the drugs between risk groups. Research shows that copper content in the tumor tissues of many cancer patients, including lung cancer, increases and is closely related to disease progression [19]. The modification of lipoacylated protein plays a unique role in copper-dependent cell death. After dissociating the Illinestimol Cu^2+^ complex, Illinestimol flows out of the cell and forms a new Illinestimol Cu^2+^ complex repeatedly, transporting Cu^2+^ from the outside of the cell to the inside, leading to the continuous accumulation of copper in the mitochondria and ultimately inducing cell apoptosis. The copper transport system helps enhance cancer chemoprevention and anti-cancer drug sensitivity [20,21]. Similar research has established that copper is associated with the resistance to platinum anti-tumor compounds, showing its potential to serve as a radiotherapy tool in combined cancer treatment [19]. Therefore, it is necessary to discuss the copper metabolism and cuproptosis mechanism and to identify related drugs for the treatment of copper metabolism diseases. Cuproptosis is a new field of research, distinct from previous mechanisms of cell death. If we can fully utilize the oxidative-reduction properties of copper as a metal and simultaneously integrate the significant advantages of multi-target and multi-pathway drug molecules, it will bring more possibilities to antitumor treatment strategies. There is no doubt that this research has some limitations. First of all, the sample size was relatively limited. To verify the modeling results, training and verification were conducted in the TCGA queue, which affects the model’s effectiveness to varying degrees. Secondly, the model could not use external verification based on the RNA-seq queue to assess the model’s reliability. Furthermore, only the four lncRNAs utilized in model construction were selected for immune infiltration analysis, and more biological experiments are needed to verify these findings.

## 4. Materials and Methods

### 4.1. Downloading and Processing of Transcriptome and Mutation Data in Clinic

The analysis data set of the RNA-seq transcriptome, including 45 normal and 445 LUAD tissues, was extracted from the TCGA (https://portal.gdc.cancer.gov/) database on 20 April 2022. Tumor somatic mutation data with clinical information from TCGA include status, survival time, age, sex, and TNM stage. The annotation of lncRNA was taken from GENCODE website (https://www.gencodegenes.org/, accessed on 15 April 2022). In addition, the genes related to cuproptosis were acquired according to a previous description, as well as other literature data [1] (Supplementary Materials S1). The approval of an ethics committee in the study was not needed, as all data were downloaded from the TCGA database and strictly complied with TCGA guidelines.

### 4.2. Production and Evaluation of LncRNA Associated with Cuproptosis

The co-expression analysis of lncRNA and cuproptosis-related genes was carried out using the “limma” package in R software (version 4.2.2) or obtaining cuproptosis-related lncRNA. If |Cor| > 0.5 and *p* < 0.001 were met, it showed a correlation. Based on the co-expression analysis results, “ggplot2”, “ggalluvial”, and “dply” packages in R were used to generate Sankey diagrams.

### 4.3. Construction of Prediction Model 

The “Caret” R package randomly allocated the samples into training and test groups. Univariate Cox regression analysis was conducted on all lncRNAs related to cuproptosis using the ‘survival’ R package, and survival data were obtained. The “glmnet” package in R software (version 4.2.2) was employed to conduct Cox and Lasso regression to avoid overfitting. The best and minimum criteria with a 10-fold cross-validation penalty (λ) was chosen. Afterward, a multivariate Cox regression analysis was used to identify the differentially expressed lncRNAs concerning cuproptosis. Subsequently, the formula used for establishing the RS model was as follows: Lasso Risk Score $=\sum_{i=1}^{n}{Coef}_{i}^{*}x_{i}$, “Coef_i_” = coefficient, and “x_i_” = the normalized count of lncRNA related to every cuproptosis. Therefore, the RS of patients could be obtained using the Lasso prognostic model.

### 4.4. Verification of RS Model

Based on the median RS and the analogous training coefficient values, all the patients were divided into two groups: high and low risk. Then Kaplan–Meier (KM) analysis was employed to determine the model’s predictive efficiency for both the test and training groups. Moreover, the degree of preciseness and diagnostic ability of the CRLPM were evaluated with the help of the survival ROC curve, the time ROC packages in R, and the area under the curve (AUC). The RS model was further validated using principal component analysis (PCA), and its results were visualized with the help of the “scatterplot 3D” package in the R software (version 4.2.2). Progression-free survival (PFS) was carried out using the “survival” and “survminer” packages in R. The C index was employed to determine the risk model’s accuracy by employing the “rms”, “dplyr”, “survival”, and “pec” R packages to validate the model and the entire queue.

### 4.5. Establishment and Evaluation of Nomograms

Univariate and multivariate Cox regression was conducted for evaluation of the risk models to determine their individual prognostic efficiencies. According to the results of the analyses mentioned above, nomograms were established using R packages “rms”, “dplyr”, “survival”, and “pec” R packages, and their accuracy was determined and assessed with the help of a calibration curve.

### 4.6. Exploring the Relationship between Prognosis RS and Clinical Stages

For the verification of the RS model’s practicality at all the clinical stages, univariate and multivariate Cox regression were employed to analyze and explore the association of RS and clinical stages to reveal the potential role in LUAD.

### 4.7. GO and KEGG Enrichment Analysis

The “limma” R package was employed for the identification of differential expression genes (DEGs) among two risk groups, and the limiting condition was determined to be log2 | fold change | > 1, False Discovery Rate (FDR) < 0.05. Due to “clusterProfiler”, “org. Hs. eg. db”, and “enrichplot” R packages, GO and KEGG enrichment analyses were used to identify various molecular processes and important signaling pathways. Gene Set Enrichment Analysis (GSEA) helped identify the pathways in the TCGA database to detect 20 pathways from both risk groups. The chosen signaling pathways identified notable differences among both groups.

### 4.8. Determination of Immune Cell Infiltration and Immunotherapy in Tumors

For the purpose of studying the association among cuproptosis-related lncRNA RS and immune cell infiltration, the R software (version 4.2.2) single sample gene set enrichment analysis (ssGSEA) algorithm function package helped analyze genomic variation, assess the invasion and role of tumor-invading immune cells, and draw a correlation heat map. To predict the immunotherapeutic response, the Tumor Immune Dysfunction and Exclusion (TIDE) algorithm was employed on the basis of the simulation of the tumor immune escape process (http://tide.dfci.harvard.edu, accessed on 15 April 2022). Therefore, the difference in immunotherapy in both risk populations was observed based on the TIDE algorithm.

### 4.9. Drug Sensitivity Evaluation

The values of IC_50_ represent the semi-inhibitory concentration of the tested antagonist. To assess the clinical significance of cuproptosis-related lncRNA in the treatment of LUAD, the IC_50_ values of chemotherapy drugs were calculated with “pRRophetic” R package and its dependencies (including “car, ridge preprocessCore, genefilter, and sva”). A total of 138 drugs were involved, including midoseline, tirolimus, tibifanil, imatinib, etc. Wilcoxon symbol analysis was employed for comparing the IC_50_ difference among prominent anti-tumor drugs in both risk groups. The “ggplot2” R package was utilized for presenting the box plot.

### 4.10. Calculation of Tumor Mutational Burden Score

Tumor mutational burden (TMB) accounts for number of cancer mutations. The LUAD sample mutation data were obtained from TCGA and assessed via the “maftools” R package. The linkage between the RS of LUAD patients and TMB was depicted using the waterfall diagram.

### 4.11. Statistical Analysis

The R programming language (version 4.0.5) in R Studio was applied to perform statistical analyses. The “limma” package in R combined RNA-seq transcriptome data downloaded from TCGA with somatic mutation data. The Pearson correlation test was employed for analysis of the association between cuproptosis-related genes and lncRNA. The “Limma” R package helped in the selection of the DEGs of cuproptosis-related lncRNA. Cox regression and survival analyses were performed using R’s “survival” and “survminer” packages. The risk ratio of univariate and multivariate analysis was evaluated using the Cox Proportional Hazards model. “ClusterProfiler” in the R package was utilized to analyze the Go and KEGG pathways. The cluster analysis heat maps were plotted using the “Pheatmap” R package. The Wilcoxon rank sum test was utilized for comparison of the quantitative differences among the two sets. KM analysis using a log-rank test helped in the evaluation of the overall survival (OS) of groups. The chi-square test helped compare the categorical data among variant combinations. *p* < 0.05 was determined to be statistically significant.

## 5. Conclusions

In general, this research adds to the already known knowledge on the occurrence and progress of LUAD tumors concerning cuproptosis and determines a risk model with four kinds of lncRNAs associated with cuproptosis. This model accurately predicts the prognosis of LUAD, explores cuproptosis biomarkers that can function in predicting LUAD prognosis, and paves the way for new diagnostic and treatment strategies for LUAD.

## Figures and Tables

**Table 1:** Characteristics of LUAD patients.

Variable	Total	Validation Set	Training Set	*p*-Value
Age				
≤65	217 (48.76%)	105 (47.3%)	112 (50.22%)	0.6011
>65	228 (51.24%)	117 (52.7%)	111 (49.78%)	
gender				
FEMALE	244 (54.83%)	121 (54.5%)	123 (55.16%)	0.9657
MALE	201 (45.17%)	101 (45.5%)	100 (44.84%)	
stage				
Stage I	238 (53.48%)	125 (56.31%)	113 (50.67%)	0.3349
Stage II	102 (22.92%)	51 (22.97%)	51 (22.87%)	
Stage III	73 (16.4%)	35 (15.77%)	38 (17.04%)	
Stage IV	24 (5.39%)	8 (3.6%)	16 (7.17%)	
unknown	8 (1.8%)	3 (1.35%)	5 (2.24%)	
T stage				
T1	152 (34.16%)	80 (36.04%)	72 (32.29%)	0.8565
T2	235 (52.81%)	115 (51.8%)	120 (53.81%)	
T3	37 (8.31%)	17 (7.66%)	20 (8.97%)	
T4	18 (4.04%)	9 (4.05%)	9 (4.04%)	
unknown	3 (0.67%)	1 (0.45%)	2 (0.9%)	
M stage				
M0	296 (66.52%)	155 (69.82%)	141 (63.23%)	0.0703
M1	23 (5.17%)	7 (3.15%)	16 (7.17%)	
unknown	126 (28.31%)	60 (27.03%)	66 (29.6%)	
N stage				
N0	286 (64.27%)	145 (65.32%)	141 (63.23%)	0.9773
N1	83 (18.65%)	42 (18.92%)	41 (18.39%)	
N2	63 (14.16%)	30 (13.51%)	33 (14.8%)	
N3	2 (0.45%)	1 (0.45%)	1 (0.45%)	
unknown	11 (2.47%)	4 (1.8%)	7 (3.14%)	

## Data Availability

The original contributions presented in this study are included in the article/Supplementary Materials. All data are available upon request from the corresponding author.

## Supplementary Materials

The following supporting information can be downloaded at: https://www.mdpi.com/article/10.3390/ph17091244/s1, Table S1: 19 genes related to cuproptosis were showed; Table S2: The information of cuproptosis-related genes and lncRNA; Table S3/S4: High/low-risk groups containing prognostic information; Table S5: The TIDE scores among high/low-risk groups.
