# Supplementary material for: Comprehensive Analysis of Characteristics of Cuproptosis-Related LncRNAs Associated with Prognosis of Lung Adenocarcinoma and Tumor Immune Microenvironment
Source: Pharmaceuticals (Basel). 2024 Sep 21;17(9):1244. doi: 10.3390/ph17091244 (PMC11435230; doi:10.3390/ph17091244)
Supplement: Supplementary file 1 [file pharmaceuticals-17-01244-s001.zip › polish.pdf]

Bullet Edits

EDITORIAL CERTIFICATE

This document certifies that the paper listed below was edited and proofread for proper English language, grammar, punctuation, spelling, and overall style by one or more than one highly qualified native speakers at Bullet Edits. All of the suggested amendments were tracked with the Microsoft Word "Track Changes" feature. Therefore, the author had the option to reject or accept each change individually.

Manuscript Title:

Comprehensive Analysis of the Characteristics of Cuproptosis-related LncRNAs Associated with Prognosis of Lung Adenocarcinoma and Tumor Immune Microenvironment

Anti-counterfeiting Code:

86b4dac4d9bcc4c52f9cd4e1f59395d0

Date Issued:

2022-09-22 21:54:40

Bullet Edits is a registered company headquartered in the UK with a global presence. We offer a range of editing, proofreading services to authors. Our Ph.D. editors are all native English speakers from the USA and UK. Authors who work with Bullet Edits are guaranteed excellent language quality and timely delivery.

BULLET EDITS LIMITED ★ COMPANY NO. 13345261 ★ SEAL ★ ENGLAND AND WALES

Address: BULLET EDITS LIMITED, 85 Great Portland Street, London,UK
Tel: UK. (+44)20457 70286 / US.(+1)312-313-9179
Web: www.bulletedits.cn/Email: info@bulletedits.cn
VAT: GB 378 9316 43
